# Efficacy and tolerability of the investigational topical cream SD-101 (6% allantoin) in patients with epidermolysis bullosa: a phase 3, randomized, double-blind, vehicle-controlled trial (ESSENCE study)

**DOI:** 10.1186/s13023-020-01419-3

**Published:** 2020-06-23

**Authors:** Amy S. Paller, John Browning, Milos Nikolic, Christine Bodemer, Dedee F. Murrell, Willistine Lenon, Eva Krusinska, Allen Reha, Hjalmar Lagast, Jay A. Barth, on behalf of the ESSENCE Study Group

**Affiliations:** 1 Northwestern University Feinberg School of Medicine, Chicago, IL USA; 2 Texas Dermatology & Laser Specialists, San Antonio, TX USA; 3 Clinical Center of Serbia, Department of Dermatology, University of Belgrade, Belgrade, Serbia; 4 EB Reference Centre, Department of Dermatology, University Hospital Necker Enfants Malades, Paris, France; 5 University of New South Wales, Sydney, NSW Australia; 6 Scioderm—An Amicus Therapeutics Company, Durham, NC USA; 7 Amicus Therapeutics, Inc, Cranbury, NJ USA

**Keywords:** Epidermolysis bullosa, Efficacy, Safety, SD-101, Wound closure, Allantoin

## Abstract

**Background:**

Epidermolysis bullosa (EB) is a rare genetic disorder that manifests as blistering and/or skin erosion. There is no approved treatment for EB; current standard of care consists of wound and pain management. SD-101 6% is a topical cream containing 6% allantoin that was developed for treating skin lesions in patients with EB. The aim of this phase 3, multicenter, randomized, double-blind, vehicle-controlled study was to assess the efficacy and safety of SD-101 6% cream versus vehicle (0% allantoin) on lesions in patients with EB.

**Methods:**

Eligible patients were ≥1 month old, had a diagnosis of EB (simplex, recessive dystrophic, or intermediate junctional) and a target wound 10–50 cm^2^ in size that was present for ≥21 days. Patients were randomly assigned to SD-101 6% cream or vehicle, which was applied topically once a day to the entire body for 3 months. Primary efficacy endpoints were time to complete target wound closure within 3 months and the proportion of patients who experienced complete target wound closure within 3 months. Post hoc subgroup analyses were conducted by patient age and in those with body surface area index of total body wound burden ≥5% at baseline.

**Results:**

In total, 169 patients were enrolled and randomly assigned to SD-101 6% (*n* = 82) or vehicle (*n* = 87). Baseline demographics and disease characteristics were similar between treatment groups. There were no statistically significant differences between treatment groups in time to target wound closure (hazard ratio, 1.004; 95% confidence interval [CI] 0.651, 1.549; *P* = 0.985) or proportion of patients with complete target wound closure within 3 months (odds ratio [95% CI], 0.733 [0.365, 1.474]; nominal *P* = 0.390). A positive trend toward faster wound closure with SD-101 6% versus vehicle was observed in patients aged 2 to <12 years and those with total body wound burden ≥5% at baseline. SD-101 6% cream was well tolerated.

**Conclusions:**

SD-101 6% cream for treatment of EB-associated lesions was not more effective than vehicle in shortening the time to complete target wound closure or achieving complete target wound closure within 3 months.

**Trial registration:**

ClinicalTrials.gov, NCT02384460; Date of trial registration, February 13, 2015; First participant enrolled, March 11, 2015.

## Introduction

Epidermolysis bullosa (EB) comprises a group of rare clinically and genetically heterogeneous disorders [1, 2], characterized by fragile skin and mucous membranes, causing blistering or erosions in response to minimal or no trauma [2]. The cutaneous and extracutaneous manifestations of EB can cause serious complications and significant morbidity, and in some cases, premature death [1]. Wound infection can lead to life-threatening sepsis, and in some EB subtypes, there is an increased risk for aggressive squamous cell carcinomas [1–4]. The chronic pain associated with EB, the hardship placed on caregivers, and the high risk for complications places a considerable psychosocial burden on both patients and their families [5–8].

The incidence of EB is estimated to be approximately 1 in 50,000 live births [9]. Most patients with EB are diagnosed with 1 of 3 types based on the ultrastructural level of skin cleavage: simplex (70%), dystrophic (20%), or junctional (10%) [2, 10, 11]. Further subclassifications are based on the affected gene and the extent of skin and mucosal disease. Diagnosis often occurs in the neonatal period, although lesions may not appear in some individuals until adolescence or later, delaying an accurate diagnosis until adulthood [6].

Despite considerable research to advance the understanding of EB pathophysiology, no treatments have been approved by regulatory authorities to date [3, 12]. Current standard of care consists of bandaging and cleaning open wounds to prevent infection, pain management, and symptomatic treatment of complications [1, 2, 13]. Consequently, there is a significant unmet need for effective medical therapies.

SD-101 6% is a topical cream containing 6% allantoin that was designed for the treatment of wounds and other skin lesions in patients with EB [14, 15]. The efficacy and safety of SD-101 6% was previously investigated in a phase 2b, multicenter, randomized, double-blind, vehicle-controlled (0% allantoin), dose-ranging study (ClinicalTrials.gov: NCT02014376) in patients with EB aged ≥6 months (*N* = 48) [14]. Results showed that, compared with vehicle, treatment with SD-101 6% resulted in a numerically higher rate of wound closure at 1 month (67% vs 41%, *P* = 0.165) and a significantly higher rate of wound closure at 2 months (82% vs 41%, *P* = 0.04) [14]. Patients in the SD-101 6% group also achieved faster target wound closure and had a greater reduction in lesional body surface area (BSA) than patients in the vehicle group. SD-101 6% was well tolerated and had a safety profile similar to that of vehicle. Herein, we report the results of the 3-month, double-blind, vehicle-controlled, phase 3 study (ESSENCE), which compared the efficacy and safety of SD-101 6% with that of vehicle in a large population of patients with EB.

## Methods

### Study design and treatment

**Fig. 1:**
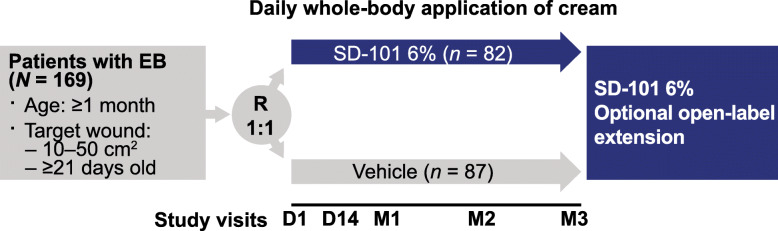
ESSENCE study design. *EB* epidermolysis bullosa; *D* day; *M* month; *R* randomization

ESSENCE (ClinicalTrials.gov NCT02384460) was a multicenter, randomized, double-blind, vehicle-controlled, phase 3 trial designed to assess the efficacy and safety of SD-101 6% versus vehicle (SD-101 0%) in patients with simplex, recessive dystrophic, or intermediate junctional EB (Fig. 1) [16]. The study was conducted between March 2015 and July 2017 in 13 countries. Patients were randomly assigned 1:1 to receive SD-101 6% or vehicle using an interactive web response system. Both SD-101 and vehicle were applied topically once daily to the entire body (including non-wounded areas) as a thin layer for 90 days. SD-101 6% is a topical cream containing 6% allantoin in an oil-in-water emulsion. The vehicle consisted of the same cream formulation as SD-101 but excluded allantoin. SD-101 and the vehicle contained the following excipients: beeswax, butylated hydroxytoluene, cetyl alcohol, citric acid, cod liver oil, lanolin oil, methylparaben, propylene glycol, propylparaben, sodium lauryl sulfate, stearyl alcohol, tetrasodium ethylenediaminetetraacetic acid, and purified water. Patient visits occurred at baseline, Day 14, Month 1, Month 2, and Month 3; those who completed the study were eligible to enroll in an open label extension study.

### Patients

Patients were eligible for study participation if they were at least 1 month old and had a diagnosis of simplex, recessive dystrophic, or intermediate junctional EB based on a skin biopsy with immunofluorescence mapping, genetic testing, or, when genetic testing was not available, medical records. In these cases, specific features of EB that allow a diagnosis to be made included hyperkeratosis of the palms and soles for EB simplex, pseudosyndactyly with esophageal strictures for recessive dystrophic EB, and enamel defects and facial granulation tissue for intermediate junctional EB [17]. Patients must have had a target wound between 10 and 50 cm^2^ in size that was present for at least 21 days based on patient history. Screening and baseline visits could be combined if patients were eligible. Patients were excluded from the study if they had clinical evidence of local infection in the target wound, used any investigational drug or systemic or topical steroidal therapy within 30 days before enrollment (inhaled steroids and ophthalmic drops containing steroids were allowed), used immunotherapy or cytotoxic chemotherapy within 60 days before enrollment, used systemic antibiotics within 7 days before enrollment, had a current or past malignancy, or had arterial or venous disorder resulting in ulcerated lesions.

### Endpoints

The primary endpoints of the study were selected in conjunction with the US Food and Drug Administration advisors, based on previous wound-healing studies. These were time to complete target wound closure within 3 months and the proportion of patients with target wound closure within 3 months. Key secondary endpoints were the proportion of patients with target wound closure at Months 1 and 2, the change in BSA index (BSAi) of lesional skin and total body wound burden at Month 3, change from baseline in patient-reported itching, and change from baseline in patient-reported pain.

### Assessments

For each patient, a target wound was selected at baseline by the investigator and measured with the SilhouetteStar™ device (ARANZ Medical, Christchurch, New Zealand), a portable, quantitative wound imaging, measurement, and documentation system. At each study visit, the SilhouetteStar™ device was used to assess complete target wound closure, defined as skin re-epithelialization without drainage.

Lesional skin, defined as skin with blisters, erosions, ulcerations, scabbing, bullae, and eschars, as well as areas that were weeping, sloughing, oozing, crusted, and/or denuded, was assessed at each visit by the same study physician. Areas of erythema and hyperpigmentation or hypopigmentation were not considered active lesions. To calculate the BSAi of lesional skin, the percentage (ranging from 0 to 100%) of the affected area was recorded for each defined body region (head/neck, upper limbs, trunk [including groin], and lower limbs), multiplied by a weighting factor, then summed for all body regions. The BSAi of total wound burden was calculated as the percentage of total body coverage of open wounds, defined as an open area on the skin (epidermal covering is disrupted).

Itching was assessed using the Itch Man Pruritus Assessment Tool at each study visit [18]. For patients 6 years of age and older, responses were recorded in patient diaries; for patients ≤5 years of age, caretakers provided responses regarding patient itching. Itching was scored on a scale from 0 (comfortable, no itch) to 4 (itches most terribly; impossible to sit still or concentrate).

Pain was assessed using the Face, Legs, Activity, Cry, Consolability (FLACC) scale for patients aged 1 month to 3 years and the Wong-Baker FACES® Pain Scale for patients aged ≥4 years [19, 20]. Each of the 5 categories in the FLACC scale is scored from 0 to 2, with a cumulative score ranging from 0 to 10. The Wong-Baker FACES® Pain Scale also ranges from 0 to 10. For both scales, higher scores indicate greater pain.

Safety was assessed via monitoring the number and incidence of adverse events (AEs), body temperature, and physical examinations. A nontreatment-emergent AE was defined as an AE that originated before the first application of study drug and did not change in severity or relationship to treatment on or after the date of the first application of study drug. A treatment-emergent AE (TEAE) was defined as an AE that occurred on or after the first date of application of study drug. If the severity of the AE or its relationship to study drug changed on or after the first application of study drug, but before the end of the study, then that AE was considered a TEAE. Relationship of AEs to treatment was assessed by the investigator. No clinical laboratory tests were performed.

### Statistics

The sample size estimation assumed that 35% of vehicle-treated patients and 60% of SD-101-treated patients would experience complete closure of their target wound at or before the 3-month follow-up visit, which corresponds to a hazard ratio of 2.127 assuming exponential hazards over time. Using these assumptions and a 1-sided overall alpha of 0.025, it was estimated that approximately 150 patients would be required (75 in each group) to provide at least 86% power to observe a difference in the time to complete target wound closure between treatment groups. Efficacy was assessed in all patients randomly assigned to a study treatment (intent-to-treat [ITT] population); safety was assessed in all patients who applied or were administered the study medication at least once (safety population).

The two primary efficacy endpoints were tested hierarchically; attainment of statistical significance for the first primary endpoint was required for continued formal testing. The first primary endpoint, time to complete target wound closure, was measured from the date of the first administration of the study drug to the date of target wound closure. Target wounds were considered closed at all time points following the first wound closure, even if it reopened. Both primary efficacy endpoints used a Cox proportional hazards model to compare treatment groups, with baseline target wound size, target wound age, and EB type as covariates. For time-to-wound-closure analyses, data were right censored at 3 months or time of withdrawal from the study.

Formal statistical testing of the second primary endpoint, the proportion of patients with target wound closure within 3 months, was performed by comparing treatment groups using the logistic regression model with baseline target wound size, target wound age, and EB type as covariates; missing values were imputed using multiple imputation methodology (missing at random).

The proportion of patients experiencing complete closure of their target wound (at 2 months or 1 month) was assessed using the same approach as the primary analysis. Changes from baseline in BSAi of lesional skin and total body wound burden were analyzed using a mixed-model repeated measures approach, including treatment, baseline BSAi, EB type, visit, and visit-treatment interaction as fixed effects. Any improvements from baseline in itching and pain were compared between treatment groups using a logistic regression model with covariates of baseline score and EB type.

Exploratory subgroup analyses were conducted to assess the consistency of treatment effects on the primary efficacy endpoints. Planned subgroup analyses include response by age at baseline (28 days to <2 years; 2 years to <12 years; 12 years to <18 years; ≥18 years). Post hoc analyses were conducted for patients with BSAi of total body wound burden ≥5 and <5% at baseline, target wound age of ≤30 or >30 days, and by EB subtype. Hazard ratios, 95% confidence intervals (CIs), and nominal *P* values were calculated for the subgroup analyses.

Incidence of skin infections in each treatment group was compared using Fisher’s exact test and summarized by treatment group (post hoc). Skin infections were defined as the following preferred terms: skin infection, wound infection, staphylococcal skin infection, skin bacterial infection, wound infection staphylococcal, folliculitis, wound infection bacterial, cellulitis, cellulitis staphylococcal, impetigo, infected skin ulcer, postoperative wound infection, and rash pustular.

## Results

### Patients

**Fig. 2:**
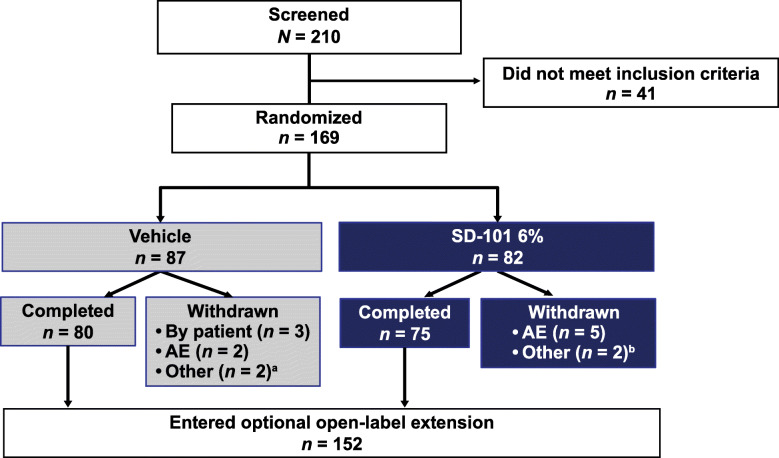
Patient disposition. *AE* adverse event. ^a^Lack of efficacy (*n* = 1); did not meet inclusion criteria/protocol deviation (*n* = 1); ^b^Elective bone marrow transplantation (*n* = 1); could not attend study visits or comply with treatment application (*n* = 1)

In total, 169 patients were enrolled from 13 countries worldwide (Australia, *n* = 11; Austria, *n* = 1; France, *n* = 19; Germany, *n* = 16; Israel, *n* = 6; Italy, *n* = 8; Lithuania, *n* = 4; Netherlands, *n* = 3; Poland, *n* = 8; Serbia, *n* = 16; Spain, *n* = 12; United Kingdom, *n* = 5; United States, *n* = 60). Eighty-two patients were randomly assigned to receive SD-101 6% and 87 were randomly assigned to receive vehicle (Fig. 2). Fourteen patients prematurely discontinued and 155 completed the study. Reasons for premature discontinuations included AEs (SD-101 6% *n* = 5, vehicle *n* = 2), withdrawal by subject (SD-101 6% *n* = 0, vehicle *n* = 3), and other (SD-101 6% *n* = 2, vehicle *n* = 2).

There were no notable differences in baseline characteristics between treatment groups (Table 1). Mean (range) age was 13.9 (0.2, 67.0) years, with approximately half (49.7%) of patients falling between the ages of 2 to <12 years of age. The SD-101 6% group had more males (59.8%) and the vehicle group had more females (55.2%). Most patients had recessive dystrophic EB.

**Table 1:** Baseline demographics and characteristics (ITT population)

	SD-101 6% *n* = 82	Vehicle *n* = 87	Total *N* = 169
Male, n (%)	49 (59.8)	39 (44.8)	88 (52.1)
Age, years			
Mean ± SD	13.8 ± 13.2	13.9 ± 13.1	13.9 ± 13.1
Median	9.0	10.5	10.0
Min, max	0.2, 67.0	0.2, 64.0	0.2, 67.0
Age group, n (%)			
1 month to <2 years	5 (6.1)	6 (6.9)	11 (6.5)
2 years to <12 years	37 (45.1)	47 (54.0)	84 (49.7)
12 years to <18 years	19 (23.2)	12 (13.8)	31 (18.3)
≥18 years	21 (25.6)	22 (25.3)	43 (25.4)
Race, n (%)			
White	69 (84.1)	72 (82.8)	141 (83.4)
Black or African American	5 (6.1)	3 (3.4)	8 (4.7)
Asian	4 (4.9)	8 (9.2)	12 (7.1)
Other/not reported	4 (4.9)	4 (4.6)	8 (4.7)
Epidermolysis bullosa type, n (%)			
Simplex	10 (12.2)	8 (9.2)	18 (10.7)
Recessive dystrophic	57 (69.5)	62 (71.3)	119 (70.4)
Intermediate junctional	15 (18.3)	17 (19.5)	32 (18.9)
Diagnosis, n (%)			
Genetic testing	36 (43.9)	35 (40.2)	71 (42.0)
Immunomapping	13 (15.9)	9 (10.3)	22 (13.0)
Medical records	33 (40.2)	43 (49.4)	76 (45.0)
Target wound size, cm^2^, mean ± SD	18.8 ± 12.1	22.0 ± 31.7	20.4 ± 24.2
Target wound age, days, mean ± SD	406.5 ± 913.8	521.0 ± 1832.0	465.4 ± 1457.4
BSAi of lesional skin, %, mean ± SD	25.8 ± 19.4	24.4 ± 19.3	25.1 ± 19.3
BSAi of total body wound burden, %, mean ± SD	12.2 ± 12.6	10.5 ± 9.1	11.3 ± 11.0
Baseline itching score^a^, mean ± SD	1.8 ± 1.21	2.0 ± 1.14	NC
Baseline pain score^b^, mean ± SD	3.1 ± 2.57	3.5 ± 2.88	NC

*BSAi* body surface area index; *ITT* intent-to-treat; *NC* not calculated; *SD* standard deviation

^a^Scale from 0 to 4

^b^Scale from 0 to 10

### Efficacy results in the overall study population

**Fig. 3:**
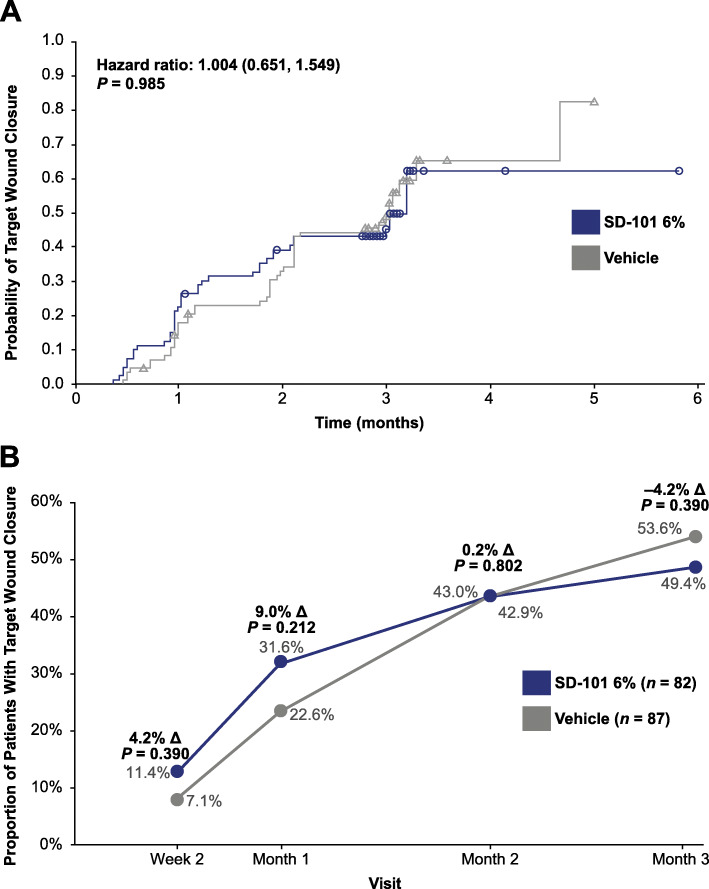
(**a**) Time to target wound closure and (**b**) proportion of patients with target wound closure by visit^a^. *ITT* intent-to-treat. ^a^ITT population (*N* = 169). Patients were censored if they did not have a response within 3 months or withdrew early before the confirmation of their target wound closing. The Kaplan-Meier curves exceed the time limit of 3 months because the study duration for a fraction of patients was more than 3 months (nominal Month 3 visit could have occurred at Month 5)

There were no statistically significant differences between the SD-101 6% and vehicle groups on the primary efficacy endpoints of time to target wound closure (hazard ratio [HR] 1.00; 95% CI 0.65, 1.55; *P* = 0.985; Fig. 3a) and the proportion of patients with complete target wound closure within 3 months (49.4% vs 53.6% in the SD-101 6% and vehicle groups, respectively; OR [95% CI], 0.73 [0.37, 1.47]; nominal *P* = 0.390; Table 2 and Fig. 3b). There were also no significant differences between the treatment groups on the key secondary endpoints, including the proportion of patients with complete target wound closure at Months 1 and 2, changes in BSAi of lesional skin or total body wound burden at Month 3, and changes in itching or pain assessments at Day 7 (Table 2) and Month 3 (data not shown).

**Table 2:** Comparison of primary endpoints and key secondary endpoints (ITT population)

	SD-101 6% *n* = 82	Vehicle *n* = 87	Nominal *P* value
Primary endpoints			
Time to complete target wound closure within 3 months, mean (SD), day	(*n* = 39) 41.6 (25.5)	(*n* = 45) 53.6 (28.6)	0.985
Complete closure of target wound within 3 months, response rate	(*n* = 79) 49.4%	(*n* = 84) 53.6%	0.390
Secondary endpoints			
Complete closure of target wound within 2 months, response rate	(*n* = 79) 43.0%	(*n* = 84) 42.9%	0.802
Complete closure of target wound within 1 month, response rate	(*n* = 79) 31.6%	(*n* = 84) 22.6%	0.212
Change in BSAi of lesional skin at Month 3, %	(*n* = 75)	(*n* = 78)	
Mean (SEM)	−4.4 (1.05)	−5.0 (1.53)	0.706
Median (range)	−2.7 (− 37.4, 18.0)	−3.0 (− 69.2, 26.5)	
Change in BSAi of total body wound burden at Month 3, %	(*n* = 75)	(*n* = 79)	
Mean (SEM)	−3.5 (0.94)	−2.3 (0.70)	0.900
Median (range)	−1.5 (−42.6, 12.5)	−1.4 (−24.1, 17.0)	
Change in itching score at Day 7	(*n* = 77)	(*n* = 79)	
Mean (SEM)	−0.5 (0.15)	−0.3 (0.14)	0.262
Median (range)	0.0 (−4, 2)	0.0 (− 4, 3)	
Change in pain score at Day 7	(*n* = 77)	(*n* = 80)	
Mean (SEM)	−0.3 (0.29)	−0.6 (0.34)	0.098
Median (range)	0.0 (−6, 10)	0.0 (−8, 10)	

*BSAi* body surface area index; *ITT* intent-to-treat; *SEM* standard error of the mean

### Subgroup analyses for target wound closure

In the subgroup of patients aged 2 to <12 years, there were no statistically significant differences in favor of the SD-101 6% group compared with the vehicle-control group for the primary efficacy endpoints and most key secondary efficacy endpoints. There was a trend toward faster wound closure with SD-101 6% versus vehicle within 3 months (HR 1.8; 95% CI 0.9, 3.5; nominal *P* = 0.085). The proportion of patients with complete target wound closure within 3 months was similar between treatment arms (55.6% versus 46.7%; OR [95% CI], 1.3 [0.5, 3.6]; nominal *P* = 0.658). However, the proportion of patients with complete target wound closure was significantly higher for the SD-101 6% group compared with the vehicle group at Week 2 (19.4% versus 2.2%; OR [95% CI], 10.9 [1.2, 99.8]; *P* = 0.034) and Month 1 (41.7% versus 20.0%; OR [95% CI], 3.4 [1.1, 10.8]; nominal *P* = 0.036).

In the subgroup of patients with ≥5% BSAi of total wound burden at baseline, no significant differences between treatment groups were observed for the primary efficacy endpoints and most key secondary efficacy endpoints. There was a trend toward faster wound closure with SD-101 6% versus vehicle within 3 months (HR 1.6; 95% CI 0.9, 2.8; nominal *P* = 0.128). Significantly more patients in the SD-101 6% group had complete target wound closure within 1 month than those in the vehicle group (35.4% versus 11.3%; odds ratio [95% CI], 3.995 [1.319, 12.104], nominal *P* = 0.014).

Although differences between treatment groups for the primary efficacy endpoints and most key secondary efficacy endpoints were also assessed by target wound age (≤30 or >30 days) and EB subtype, these analyses did not provide significant findings (data not shown).

### Safety

SD-101 6% was well tolerated with a safety profile similar to that of vehicle (Table 3). Overall, pruritus that occurred or worsened after the first administration of either treatment was the most common TEAE, occurring in 9 patients in the SD-101 6% arm and 8 patients in the vehicle arm (Table 3). Skin infections of interest, which comprised 13 skin infection-related preferred terms, were significantly less frequent in the SD-101 6% arm compared with the vehicle arm (18% versus 33.3%; *P* = 0.026) (Table 3).

**Table 3:** Summary of treatment-emergent adverse events^a^ (safety population)

n (%)	SD-101 6% *n* = 82	Vehicle *n* = 87
Overall Summary		
Any AE	71 (86.6)	61 (70.1)
Any serious AE	4 (4.9)	8 (9.2)
Any AE leading to death	0	1 (1.1)^b^
Any AE leading to discontinuation	5 (6.1)	3 (3.4)
AEs Occurring in ≥ 5% of Patients in Either Treatment Arm		
Worsening pruritus	9 (11.0)	8 (9.2)
Nasopharyngitis	11 (13.4)	3 (3.4)
Pyrexia	7 (8.5)	9 (10.3)
Wound infection	6 (7.3)	5 (5.7)
Upper respiratory tract infection	4 (4.9)	9 (10.3)
All skin infection	3 (3.7)	9 (10.3)
Staphylococcal skin infection	1 (1.2)	7 (8.0)
AEs of Special Interest		
Patients who had skin infection^c^	15 (18.3)	29 (33.3)^d^

*AE* adverse event

^a^Defined as AEs that began or changed in severity or relationship to treatment on or after the date of the first dose of study medication

^b^One case of a non-treatment-related death occurred on Day 62 after initiation of treatment owing to cardiac disorders and cardiopulmonary failure

^c^Patients were considered to have a skin infection if meeting one of the following preferred terms: skin infection, wound infection, staphylococcal skin infection, bacterial skin infection, staphylococcal wound infection, folliculitis, bacterial wound infection, cellulitis, staphylococcal cellulitis, impetigo, infected skin ulcer, postoperative wound infection, or pustular rash

^d^*P* = 0.026 based on Chi-square test

AEs leading to study discontinuation and serious AEs were uncommon in both treatment groups (Table 3). AEs considered by the investigator to be treatment-related were similar in both treatment groups (Table 4). Pruritus was the most common treatment-related AE, occurring in 6 patients in the SD-101 6% arm and 5 in the vehicle arm.

**Table 4:** Treatment-related adverse events^a^ (safety population)

n (%)	SD-101 6% *n* = 82	Vehicle *n* = 87
Any treatment-related AE	15 (18.3)	19 (21.8)
Treatment-Related AEs Occurring in ≥2% of Patients in Either Treatment Arm		
Pruritus (localized or generalized)	6 (7.3)	5 (5.7)
Wound	2 (2.4)	2 (2.3)
Blister	2 (2.4)	2 (2.3)
Urticaria	2 (2.4)	0
Dry skin	2 (2.4)	0
Generalized pruritus	2 (2.4)	0
Staphylococcal skin infection^b^	0	4 (4.6)
Skin infection^b^	0	3 (3.4)
Dermatitis^c^	0	2 (2.3)
Maculopapular rash^d^	0	2 (2.3)

*AE* adverse event

^a^AEs were deemed unrelated, possibly, probably, or definitely related to treatment by the investigator

^b^Staphylococcal skin infection was confirmed with either microbiology sample or antibiotic treatment, and skin infection included all unspecified skin infection

^c^Described as dermatitis without pruritus or pain (*n* = 1) and lumber area skin inflammation (*n* = 1)

^d^Patients with maculopapular rash are different from those with dermatitis

One death was reported in the vehicle treatment arm. The patient was a 10-year-old male with intermediate junctional EB who was withdrawn from the study by his father on day 28. On day 38, the child was diagnosed with influenza, which was ongoing on day 62, when he died from cardiopulmonary failure. The death was considered by the investigator to be unrelated to treatment.

## Discussion

EB is a devastating inherited disorder for which the greatest unmet treatment needs include effective wound healing and prevention, and control of pain and itch [21]. SD-101 6% was developed as a topical therapy for the treatment of EB and is designed to deliver a high concentration (6%) of allantoin to lesional skin in a highly stable, soluble form. In preclinical studies, allantoin was shown to have multiple wound-healing effects, including anti-inflammatory and antimicrobial activity as well as promoting tissue formation and differentiation, specifically in stimulating collagen deposition and epithelialization [22, 23]. Allantoin is excreted in maggot urine, which may explain the effectiveness of maggot therapy in wound healing [24]. In humans, allantoin is produced in the amniotic membranes only, which themselves have been used to treat wounds of patients with recessive dystrophic EB [25]. Evidence for the clinical benefit of SD-101 6% was observed in the phase 2b study, with a numerically higher rate of wound closure after 1 month compared with vehicle [14]. Data from this study provided the basis for the present phase 3 trial, which assessed the efficacy and safety of SD-101 6% over 3-months’ duration in a large, heterogeneous EB patient population with a broad range of demographic and disease characteristics. However, no statistically significant differences were observed in primary or key secondary efficacy endpoints in the intent-to-treat population (*n* = 169). These observations are in contrast to results from the phase 2b trial, in which SD-101 6% showed clinical benefit compared with vehicle [14]. However, in the phase 2b trial, the primary endpoint was assessed earlier (at Month 1), which may have contributed to the clinical benefit reported in that study [14].

Given that children with EB experience functional impairments that can substantially decrease quality of life [26], and children aged 2 to <12 years comprised half (*n* = 84) of the overall study population, planned subgroup analyses were conducted in patients aged 2 to <12 years. These analyses demonstrated a trend toward earlier wound closure with SD-101 6% at Week 2 and Month 1, and the proportion of patients achieving target wound closure numerically favored SD-101 6% at Month 1, compared with vehicle. These observations suggest that SD-101 6% may promote faster wound closure compared with vehicle in patients aged 2 to <12 years. Notably, treatment difference (versus vehicle) only reached statistical significance at Week 2 and at Month 1. Because placebo response would be expected to be highest in patients with mild disease, efficacy was also evaluated in a post hoc analysis of the subgroup of patients with ≥5% BSAi of total body wound burden at baseline. Similar to the subgroup analyses based on age, a positive trend toward wound healing at earlier time points was noted in patients with ≥5% BSAi of total body wound burden at baseline who received SD-101 6% compared with vehicle. Overall, SD-101 6% was generally well tolerated, with TEAEs occurring at generally similar frequencies across treatment groups. Two patients who received SD-101 6% experienced urticaria compared with none in the vehicle arm, suggesting the possibility of a hypersensitivity reaction to SD-101 6%.

The lack of a statistically significant difference in wound closure between SD-101 and vehicle at Month 3 could be due to a higher-than-expected placebo response rate, which increased over time (manuscript submitted as a companion paper). A high rate of therapeutic success in the vehicle group appears to be a frequent effect in controlled clinical studies of patients with EB [27–31], and may have contributed to the lack of a statistically significant difference in the present study. It is possible that one or more components of the vehicle control cream had therapeutic effects. The vehicle used in the present study contained lanolin oil and cod liver oil, both of which have been shown to promote skin healing [32, 33], and therefore could have contributed to the higher-than-expected levels of wound closure observed in the control group. Notably, petroleum, once considered an “inert” moisturizer, was recently shown to robustly modulate antimicrobials and epidermal differentiation [34], further supporting the premise that vehicles alone may have therapeutic benefit. These observations support the benefits of moisturizing skin care for the management of EB, which have not been formally studied. In addition, daily application of a topical cream to the entire body may have contributed to the improvements observed in the vehicle arm, as the degree of skin care that occurred during the trial may have been greater than the skin care routine normally practiced at home.

Several therapeutic approaches have been evaluated for the treatment of EB. However, most studies were not randomized or controlled, and few have shown any meaningful clinical efficacy [35]. Nonetheless, ongoing research holds promise for new therapeutic strategies. Findings from our study may have important implications for the design of future clinical studies in patients with EB. A placebo run-in period of 3 months’ duration preceding randomization should be considered in future EB study designs, allowing the exclusion of “early responders” or those who respond to proper wound care and components of vehicle controls (e.g., lanolin, cod liver oil) prior to randomization to trial treatment. However, this approach may be limited by low enrollment numbers owing to the rarity of EB, as obtaining enough patients to attain sufficient power to detect efficacy is challenging even with a global enrollment strategy. In addition, given that patients with EB have multiple wounds that fluctuate and recur, future studies should include whole patient assessments, such as the validated EBDASI, iScorEB, and QOLEB scores. The possibility that our study was confounded by a high rate of vehicle response is supported by the post hoc subgroup analysis in patients with BSAi of total wound burden ≥5%. When patients with the mildest disease (BSAi <5%) were removed from the analysis, numerically greater differences between SD-101 6% and vehicle were observed. Similarly, patients aged <2 years who were treated with vehicle showed a numerically higher response than other age groups (manuscript submitted as a companion paper); when this age group was excluded, greater improvements with SD-101 6% compared with vehicle were apparent in patients aged 2 to <12 years of age.

## Conclusions

ESSENCE included a broad representation of EB subtypes and is the largest randomized, controlled clinical trial of an investigational drug in patients with EB completed to date. In this study, no significant differences between SD-101 6% and vehicle were observed for the primary efficacy endpoints of time to complete target wound closure or the proportion of patients with complete target wound closure within 3 months. A positive trend toward faster wound healing with SD-101 6% at earlier time points (within 1 month) was noted for subgroups of patients between 2 and <12 years of age and those with ≥5% BSAi of total body wound burden at baseline. SD-101 6% was generally well-tolerated with a safety profile similar to that of vehicle.

## Supplementary information

**Additional file 1:.** ESSENCE individual patient data. Contains individual de-identified participant data regarding the target wound from the current study.

**Additional file 2:.** ESSENCE study site information. Presents study investigators, country, and patient numbers for all study sites.

## Data Availability

Individual de-identified participant data regarding the target wound from the current study are published as supplemental material.

## Abbreviation

AE: adverse event
BSAi: body surface area index
CI: confidence interval
D: day
EB: Epidermolysis bullosa
FLACC: Face, Legs, Activity, Cry, Consolability scale
ITT: intent-to-treat
M: month
NC: not calculated
RDEB: recessive dystrophic epidermolysis bullosa
SD: standard deviation
SE: standard error
SEM: standard error of the mean
